# Long-term oral appliance therapy in obstructive sleep apnea syndrome: a controlled study on dental side effects

**DOI:** 10.1007/s00784-012-0737-x

**Published:** 2012-05-06

**Authors:** M. H. J. Doff, K. J. Finnema, A. Hoekema, P. J. Wijkstra, L. G. M. de Bont, B. Stegenga

**Affiliations:** 1 Department of Oral and Maxillofacial Surgery, University Medical Center Groningen, University of Groningen, Hanzeplein 1, P.O. Box 30.001, 9700 RB Groningen, The Netherlands; 2 Department of Orthodontics, University Medical Center Groningen, University of Groningen, Groningen, The Netherlands; 3 Department of Oral and Maxillofacial Surgery, Tjongerschans Hospital, Heerenveen, The Netherlands; 4 Department of Home Mechanical Ventilation, University Medical Center Groningen, University of Groningen, Groningen, The Netherlands

**Keywords:** CPAP, Obstructive sleep apnea syndrome, Oral appliance, Side effects, Study models, Therapy

## Abstract

**Objectives:**

This study aimed to assess possible dental side effects associated with long-term use of an adjustable oral appliance compared with continuous positive airway pressure (CPAP) in patients with the obstructive sleep apnea syndrome and to study the relationship between these possible side effects and the degree of mandibular protrusion associated with oral appliance therapy.

**Materials and methods:**

As part of a previously conducted RCT, 51 patients were randomized to oral appliance therapy and 52 patients to CPAP therapy. At baseline and after a 2-year follow-up, dental plaster study models in full occlusion were obtained which were thereupon analyzed with respect to relevant variables.

**Results:**

Long-term use of an oral appliance resulted in small but significant dental changes compared with CPAP. In the oral appliance group, overbite and overjet decreased 1.2 (±1.1) mm and 1.5 (±1.5) mm, respectively. Furthermore, we found a significantly larger anterior–posterior change in the occlusion (−1.3 ± 1.5 mm) in the oral appliance group compared to the CPAP group (−0.1 ± 0.6 mm). Moreover, both groups showed a significant decrease in number of occlusal contact points in the (pre)molar region. Linear regression analysis revealed that the decrease in overbite was associated with the mean mandibular protrusion during follow-up [regression coefficient (*β*) = −0.02, 95 % confidence interval (−0.04 to −0.00)].

**Conclusions:**

Oral appliance therapy should be considered as a lifelong treatment, and there is a risk of dental side effects to occur.

**Clinical relevance:**

Patients treated with the oral appliance need a thorough follow-up by a dentist or dental-specialist experienced in the field of dental sleep medicine.

## Introduction

Obstructive sleep apnea syndrome (OSAS) is characterized by repetitive episodes of pharyngeal collapse with increased airflow resistance during sleep [1] and is often accompanied by extensive snoring. OSAS is associated with excessive daytime sleepiness, (sexual) dysfunction, neurocognitive deficits, and higher rates of cardiovascular and cerebrovascular morbidity and mortality [2–5]. In the North American population, OSAS affects approximately 4 % of the male and 2 % of the female adults [4]. The severity of the disorder is usually expressed by the apnea–hypopnea index (AHI), i.e., the mean number of apneas and hypopneas per hour of sleep and is classified as mild (AHI 5–15), moderate (AHI 15–30), or severe (AHI >30) [6]. Standard treatment with continuous positive airway pressure (CPAP) is highly efficacious for OSAS but adherence to the treatment limits its overall effectiveness [7]. Oral appliance therapy is a viable alternative in the treatment of OSAS, especially in the mild and moderate cases and in patients unwilling or unable to tolerate CPAP [8]. Generally, oral appliances aim at enlarging the upper airway during sleep by holding the mandible in a forward and downward position [9].

Mild and “transient” side effects are commonly reported in the initial period of oral appliance therapy and include tooth pain, temporomandibular joint pain, myofascial pain, dry mouth, excessive salivation, and gum irritation [10–18]. In several studies, dental side effects related to long-term use of an oral appliance have been studied with study model analysis [16, 19–22]. However, most of these studies were retrospective, comprised small study samples, did not include a control group or only included snorers or mild and moderate OSAS patients. Furthermore, all studies except for two [21, 22] evaluated the effects of an oral appliance that was nonadjustable and fixed the mandible in a predefined position at 50–75 % of the maximum mandibular protrusion. Therefore, the relationship between the amount of mandibular protrusion during follow-up and the extent of dental side effects is an aspect that needs further study.

The objectives of this parallel controlled follow-up study were to assess:

1. The occurrence of dental side effects following long-term (2 years) oral appliance therapy compared with CPAP in a prospective study in patients with mild-to-severe OSAS
2. The relationship between the mean mandibular protrusion and the degree of dental side effects to occur

## Materials and methods

### Patient selection

The efficacy of an oral appliance in the treatment of OSAS, compared with CPAP, has been evaluated in a previously executed randomized controlled trial [23]. The materials and methods of this specific study are briefly summarized below. All patients were recruited through the Department of Home Mechanical Ventilation of the University Medical Center Groningen, the Netherlands. Subjects over 20 years of age and diagnosed with OSAS (AHI >5) based on polysomnography [6] were eligible. If patients fulfilled predefined medical, psychological, and dental inclusion criteria, they were selected for the study and subsequently randomized for either oral appliance (*n* = 51) or CPAP therapy (*n* = 52).

**Fig. 1:**
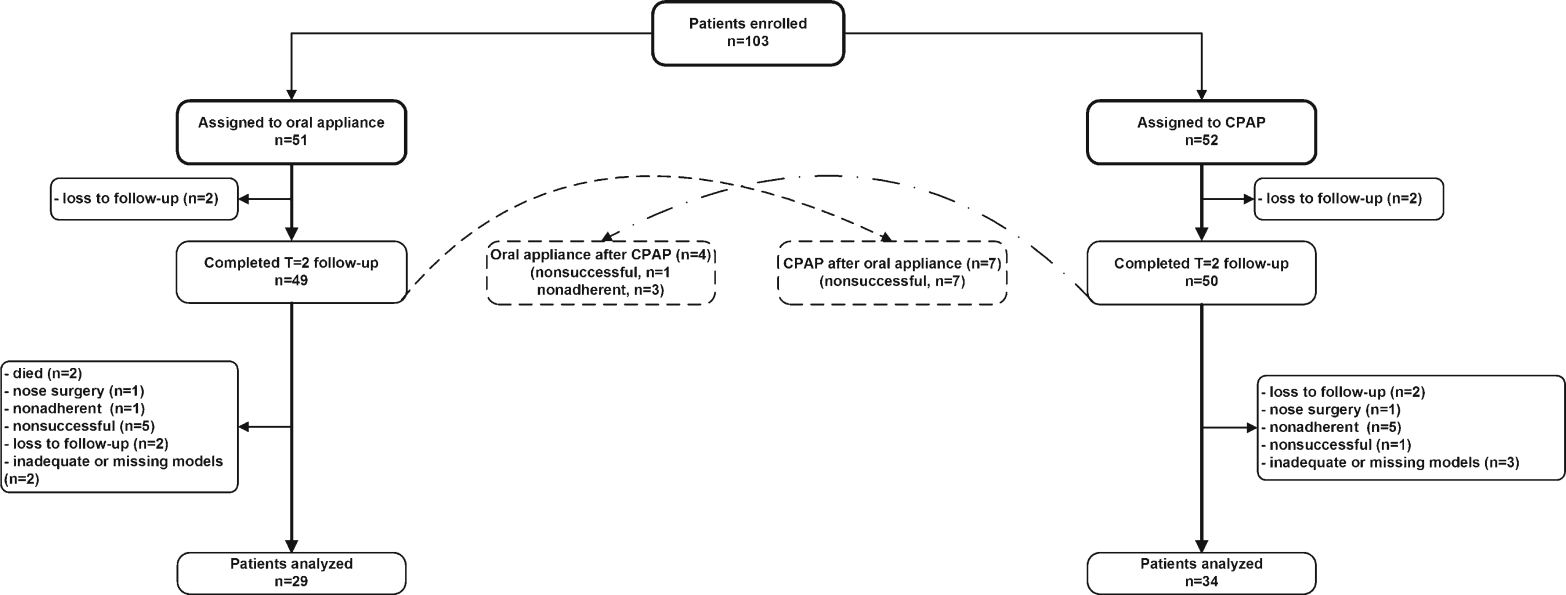
Flow diagram of the patient selection procedure. Patients who discontinued treatment for any reason were considered nonadherent to treatment. Treatment was considered successful when the apnea–hypopnea index was <5 or showed substantial reduction in the index of at least 50 % from the baseline value to a value of <20 in a patient without symptoms while undergoing therapy. Patients not meeting these criteria were considered nonsuccessful

For the present study, we assessed dental side effects in the group of patients treated with an oral appliance compared to those treated with CPAP. Patients considered *nonsuccessful* or *nonadherent* [23] were offered to switch to the alternative therapy at any time during the follow-up. Patients that switched from the oral appliance therapy to CPAP or vice versa were excluded from analysis. Furthermore, patients were excluded if they received upper airway surgery during follow-up or if the oral appliance was used <5 nights a week or <5 h per night. Details of patient selection criteria for our study are provided in Fig. 1.

The present study was approved by the Groningen University Medical Center's Ethics Committee (METc2002/032). A written informed consent was obtained from each patient before enrolment.

### Study design

At baseline, the patients had been subjected to a polysomnographic evaluation, based on which they were classified as having non-severe (AHI 5–30) or severe (AHI >30) OSAS. From all patients, dental plaster study models were obtained at baseline and after 2 years of treatment to determine variables related to the dental morphology and the (dental) occlusion.

The oral appliance used in this study (Thornton Adjustable Positioner, Airway Management Inc., Dallas, TX, USA) consisted of two separate parts, fixing the patient's mandible in a forward and downward position. This type of oral appliance is often referred to as a mandibular repositioning appliance. By turning a propulsion screw that was incorporated anteriorly in the appliance, patients could adjust the mandibular advancement with 0.2 mm increments with each turn. The maximum range of mandibular protrusion was first determined with a George-Gauge™ (H-Orthodontics, Michigan City, IN, USA). When initiating oral appliance therapy, the mandible was set at 50 % of the patient's maximum protrusion. After having accustomed to this protrusive position during a 2-week period, the patients were allowed to further adjust the oral appliance during a 6-week period. When OSAS symptomatology (e.g., snoring, excessive daytime sleepiness, apneas and/or hypopneas) appeared to persist, patients were instructed to advance the mandible each night with one to two increments (i.e., 0.2–0.4 mm). This adjustment period had extended until symptoms were adequately improved (e.g., no signs of apneas, hypopneas, and snoring) or until further protrusion of the mandible resulted in discomfort. The mean mandibular protrusion during the follow-up period (expressed as percentage of the maximum mandibular protrusion) was used for further analysis. The vertical dimension of the oral appliance was kept constant during the entire follow-up period. Both mandibular protrusion and mouth opening (including the vertical overbite) imposed by the oral appliance were measured with a digital sliding calliper. These measurements were carried out at baseline, after 2 months, 1 year, and 2 years of treatment. At these time-points, also other clinical measurements (weight, length, and neck circumference) were carried out. At baseline and after 2 years of treatment, dental plaster study models in full occlusion were obtained for all patients.

CPAP titration was performed during an afternoon nap. This technique, aimed at abolishing all signs of apneas, hypopneas, and snoring, has been shown to be an appropriate procedure for the effective titration of CPAP [24].

Following CPAP and oral appliance adjustment, an 8-week follow-up period was arranged that allowed for habituation and, if necessary, adjustment of CPAP or the oral appliance. After this period, a second polysomnographic study was performed. If polysomnography indicated an AHI ≥5, CPAP or the oral appliance was further adjusted by the physician (not during polysomnography). A third polysomnographic study was performed 4 weeks after that adjustment.

Treatment was considered successful when the AHI either was <5 or showed “substantial reduction,” [23] defined as a reduction in the index of at least 50 % from the baseline value to a value of <20 in a patient who had no symptoms while using therapy. Patients for whom oral appliance or CPAP therapy was successful continued this treatment. If one of both treatments was not successful at any time during the follow-up period, patients were offered the alternative therapy (CPAP or oral appliance, respectively), which was titrated as described above. After a 2-year follow-up period, all patients were subjected to a final polysomnographic evaluation. Patients were always encouraged to contact our clinic between regular follow-up appointments when problems were faced concerning the oral appliance, CPAP device, or treatment effect.

### Study model analysis

At baseline and after 2 years of treatment, dental plaster study models in full occlusion, based on alginate impressions of the upper and lower dental arches, were obtained from all patients to determine clinically relevant variables related to the patient's occlusion [16, 21, 25]. The intermaxillary relationship between the upper and lower arch was recorded with the vinyl polysiloxan registration material Exabite II NDS^TM^ (GC America Inc, Alsip, Il, USA) in maximal occlusion. Models were also placed and rimmed into maximal occlusion in order to perform measurements with a digital sliding calliper with a 0.01-mm resolution [26]. All measurements were carried out on models in full occlusion, mounted in an articulator (Artex®, Girrlach Dental, Koblach, Germany) with a bilateral sagittal condylar inclination of 32.5° and Bennett angle of 17.5°. The degree of mandibular protrusion and mouth opening, associated with wearing an oral appliance, was also measured with a digital sliding calliper while the oral appliance was fixed on the models in the therapeutic position, which were subsequently mounted in the articulator. Measurements were performed twice by one observer (K.F.) who was blinded for the patient's treatment. For continuous variables, the mean of both measurements was used for further analysis.

Anterior overjet and overbite were calculated as the mean of the overjet or overbite measured at both maxillary central incisors. Anterior overjet was defined as the horizontal distance from the mesial end of the incisal edge of the upper central incisor to the labial plane of the lower central incisor. Anterior overbite was defined as the vertical distance of the incisal edge of the lower central incisor to the horizontal projection of the incisal edge of the upper central incisor on the labial plane of the lower central incisor. The difference (baseline versus follow-up) in anterior–posterior relationship was measured at the buccal sites of the first molars. In order to obtain reproducible measurements, the position of the buccal groove of the mandibular first molar was marked on the buccal site of the maxillary antagonist. Negative values are related to a mesial shift of the dentition in the mandible and positive values are related to a distal shift of the dentition in the mandible. If the first permanent molars were missing, the second permanent molars were used for measurements.

Angle's classification system [27] of malocclusion was used to identify the anterior–posterior relationship of the upper and lower first molars and upper and lower cuspids and was classified as class I (neutrocclusion), class II (distocclusion), and class III (mesiocclusion). Angle classifications were determined at baseline and follow-up for the left and right molar occlusion (if the upper and lower first molars were present; Table 2) and for the left and right cuspid occlusion (if the upper and lower cuspid were present; Table 3). When models were missing, damaged, or if too many teeth were missing for adequate classification, the patient was listed as *indefinable*.

Occlusal contact points (relationship between maxillary and mandibular teeth in maximal occlusion) were made visible using articulation paper directly on the models. Biting forces were not applied in a standardized way as we did not have the disposal of such equipment. Subsequently, the number of teeth in contact was determined, not the size of the contact points. The number of occlusal contact points was determined for the (pre)molar region and for the cuspid–incisor region of the maxillary model.

The left and right transversal relation between the maxillary and mandibular teeth in the (pre)molar region was determined as normal, end-to-end, or crossbite. If the transversal relation of one or more teeth at the (pre)molar region changed after the follow-up period, this was recorded.

Crowding was defined (visually) as an altered tooth position as a result of inadequate space in the alveolar arch and was scored as increased, decreased, or no change after the follow-up period. Evaluation of interproximal spaces (diastemas) was done at baseline and after follow-up for both arches and was classified as unchanged, increased, or decreased. Interproximal spaces at follow-up that resulted from tooth extractions during the follow-up period were not counted. Dummies incorporated in fixed bridges were included in all analyses but were not counted as present permanent teeth.

### Statistical analysis

Statistical analyses were performed using the Statistical Package for the Social Sciences (version 16.0, SPSS Inc., Chicago, IL, USA). All variables were normally distributed and their means and standard deviations were reported. The AHI of the oral appliance and CPAP patients at baseline was distributed normally after logarithmic transformation. To compare outcomes between continuous variables at baseline and follow-up, paired Student's *t* tests were performed. For comparing outcomes for continuous variables between the oral appliance and CPAP group, unpaired Student's *t* tests were performed. For comparing categorical variables within and between both treatment groups, the Fischer's exact test was applied. Linear regression analysis was used to determine the relationship between possible dental side effects and other therapy- or patient-related variables during the follow-up period (e.g., mandibular protrusion and degree of overjet (OJ) and overbite (OB). Regression coefficients (*β*) and their 95 % confidence intervals (CI) are reported. Other analyses were exploratory. A significance level *α* of 0.05 was predefined in all cases.

## Results

For analysis, 29 and 34 patients were included in the oral appliance group and the CPAP group, respectively, after 2 years of treatment (Fig. 1). The mean follow-up period was 2.3 (± 0.2) years in the oral appliance group (range 2.1–3.1 years) and 2.4 (±0.3) years in the CPAP group (range 2.1–3.2 years). The mean mandibular protrusion during the follow-up period was 79 (±19) % of the patient's maximum protrusion. The numbers of nights and hours per night that both therapies were used did not significantly differ between both treatment groups (Table 1).

**Table 1:** Baseline characteristics, therapeutic use, and cast analysis of patients who completed the 2-year follow-up

Variable	Oral appliance (*n* = 29)^a^	CPAP (*n* = 34)^a^	Difference (*p* value)^c^
Age (years)	49.7 ± 8.9.	50.6 ± 10.1	NS
Male/female ratio	22/7	32/2	NS^d^
Apnea–hypopnea index (no./h)	35.6 ± 22.3	44.2 ± 27.9	NS
Body mass index (kg/m^2^)	31.4 ± 5.7	33.7 ± 5.7	NS
Therapeutic use			
Nights per week	6.9 ± 0.4	6.7 ± 1.1	NS
Hours per night	7.1 ± 0.8	6.7 ± 1.3	NS
Number of teeth			
Upper arch	12.7 ± 1.5	13.1 ± 1.8	NS
Lower arch	13.1 ± 1.4	13.0 ± 1.5	NS
Occlusal contact points cuspid–incisor region (no.)			
Baseline	2.5 ± 1.7	3.2 ± 2.0	NS
Follow-up	2.2 ± 1.8	3.0 ± 1.9	NS
Difference (*p* value)^b^	NS	NS	
Occlusal contact points (pre)molar region (no.)			
Baseline	6.8 ± 2.6	6.9 ± 2.6	NS
Follow-up	5.1 ± 2.3	6.4 ± 2.3	NS
Difference (*p* value)^c^	*p* < 0.00	*p* = 0.03	
Delta overbite (mm)	−1.2 ± 1.1	−0.1 ± 0.6	*p* < 0.00
Delta overjet (mm)	−1.5 ± 1.5	−0.2 ± 0.7	*p* < 0.00
Anterior–posterior movement (mm)	−1.3 ± 1.5	−0.1 ± 0.6	*p* < 0.00

*CPAP* continuous positive airway pressure, *NS* not significant

^a^Values are means ± standard deviations

^b^Unpaired *t* test

^c^Paired *t* test

^d^Fischer's exact test

### Study model analysis

Regarding baseline characteristics of the patients included in the analysis, (Table 1), no significant differences were found between the oral appliance and CPAP group. A significant decrease in overbite (−1.2 ± 1.1 mm; *p* < 0.00) and overjet (−1.5 ± 1.5 mm; *p* < 0.00) was found in the oral appliance group compared to the CPAP group as well as a significantly (*p* < 0.00) larger anterior–posterior change in occlusion (−1.3 ± 1.5) compared to the CPAP group (−0.1 ± 0.6 mm) (Table 1). For the oral appliance group, linear regression analysis revealed a significant association between the change in overbite and the amount of mandibular protrusion (*β*= − 0.02; 95 % CI −0.04 to 0.00). We did not find a significant association between the baseline OJ or OB and the change in OJ or OB after long-term oral appliance or CPAP use.

When evaluating the number of occlusal contact points between the upper and lower dental arch in the cuspid–incisor region, there was no tendency towards the occurrence of an open bite in the frontal region in either the oral appliance or the CPAP group. We found a significant decrease in the number of occlusal contact points in the (pre)molar region in both the oral appliance (*p* < 0.00) and CPAP group (*p* = 0.03) after 2 years of treatment (Table 1).

Transversal relation of the teeth in the (pre)molar region did change in eight patients in the oral appliance group and in two patients in the CPAP group after 2 years of treatment. In four patients from the oral appliance group, the bilateral transversal relation changed from normal to end-to-end and in one patient only the right side transversal relation changed from normal to end-to-end. In one patient, a bilateral crossbite changed to a complete reversed transversal relation (maxillary inner bite). However, this patient was familiar with a midface hypoplasia and a class III malocclusion before starting oral appliance therapy. Furthermore, in four patients, the bilateral transversal relation changed from end-to-end to a bilateral crossbite. In the CPAP group, one patients' bilateral transversal relation changed from normal to a bilateral crossbite and one changed from a normal bilateral transversal relation to a bilateral end-to-end relation.

For the CPAP group, left side molar occlusion could be assessed at baseline in 25 patients and right side molar occlusion in 29 patients (Table 2). After 2 years of treatment, a change in right side molar occlusion was observed in three (10 %) patients and in the left side molar occlusion also in three patients (12 %). Furthermore, left side cuspid occlusion was determined in all 34 patients and right side cuspid occlusion for 33 patients (Table 3). After 2 years of treatment, a change in right side cuspid occlusion was verified in four patients (12 %) and in one patient (3 %) for the left side cuspid occlusion.

**Table 2:** Molar occlusion at baseline and follow-up review

Angle’s classification	Oral appliance group (*n* = 29)				CPAP group (*n* = 34)			
	Molar occlusion right side		Molar occlusion left side		Molar occlusion right side		Molar occlusion left side	
	Baseline	Follow-up	Baseline	Follow-Up	Baseline	Follow-Up	Baseline	Follow-Up
Class I	*n* = 7	Unchanged (*n* = 4)	*n* = 6	Unchanged (*n* = 4)	*n* = 9	Unchanged (*n* = 7)	*n* = 6	Unchanged (*n* = 4)
		Class III (3)		Class II (*n* = 1)		Class II (*n* = 1)		Class II (*n* = 1)
				Indefinable (*n* = 1)		Indefinable (*n* = 1)		Indefinable (*n* = 1)
Class II	*n* = 14	Unchanged (13)	*n* = 14	Unchanged (*n* = 11)	*n* = 16	Unchanged (*n* = 15)	*n* = 15	Unchanged (*n* = 14)
		Class III (*n* = 1)		Class I (*n* = 2)		Class I (*n* = 1)		Indefinable (*n* = 1)
				Class III (*n* = 1)				
Class III	*n* = 0		*n* = 1	Unchanged (*n* = 1)	*n* = 4	Unchanged (*n* = 3)	*n* = 4	Unchanged (*n* = 2)
						Class I (*n* = 1)		Class I (*n* = 2)
Indefinable	*n* = 8		*n* = 8					

*CPAP* continuous positive airway pressure

**Table 3:** Cuspid occlusion at baseline and follow-up review

Angle’s classification	Oral appliance group (*n* = 29)				CPAP group (*n* = 34)			
	Cuspid occlusion right side		Cuspid occlusion left side		Cuspid occlusion right side		Cuspid occlusion left side	
	Baseline	Follow-up	Baseline	Follow-up	Baseline	Follow-up	Baseline	Follow-up
Class I	*n* = 9	Unchanged (*n* = 5)	*n* = 9	Unchanged (*n* = 4)	*n* = 13	Unchanged (*n* = 12)	*n* = 8	Unchanged (*n* = 4)
		Class III (*n* = 4)		Class III (*n* = 5)		Class II (*n* = 1)		Class I (*n* = 1)
								Indefinable (*n* = 3)
Class II	*n* = 18	Unchanged (*n* = 16)	*n* = 18	Unchanged (*n* = 15)	*n* = 19	Unchanged (*n* = 16)	*n* = 24	Unchanged (*n* = 22)
		Class III (*n* = 2)		Class I (*n* = 3)		Class I (*n* = 2)		Class I (*n* = 1)
						Class III (*n* = 1)		Indefinable (*n* = 1)
Class III	*n* = 1	Unchanged (*n* = 1)	*n* = 2	Unchanged (*n* = 2)	*n* = 1	Unchanged (*n* = 1)	*n* = 2	Unchanged (*n* = 2)
Indefinable	*n* = 1				*n* = 1			

*CPAP* continuous positive airway pressure

For the oral appliance group, both left and right side molar occlusion could be determined at baseline in 21 patients (Table 2). After 2 years of treatment, a change in right side molar occlusion was observed in four patients (20 %) and in the left side molar occlusion also in four patients (20 %). Furthermore, left side cuspid occlusion was assessed for all 29 patients and right side cuspid occlusion for 28 patients (Table 3). After 2 years of treatment, a change in right side cuspid occlusion was observed in six patients (21 %) and in eight patients (28 %) for the left side cuspid occlusion.

No significant differences in crowding were observed after 2 years of treatment for both treatment groups. In the oral appliance group, no differences were found in crowding for the upper dental arch while one patient showed less crowding for the lower dental arch. In the CPAP group, one patient showed less crowding for the upper dental arch, one showed less crowding, and one showed more crowding for the lower dental arch.

No significant differences in interproximal spaces were found for the upper and lower arch between the oral appliance and the CPAP group. In the oral appliance group, an increase in interproximal spaces in the upper dental arch was found in two patients and a decrease in two patients. For the lower dental arch, an increase in interproximal spaces was found in seven patients. In the CPAP group, an increase in interproximal open spaces of the upper dental arch was found in one patient, whereas for the lower dental arch, interproximal spaces were increased in four patients.

## Discussion

To our knowledge, this is the first study in which changes in dental morphology as a result of long-term oral appliance therapy are evaluated in a controlled study concerning mild-to-severe OSAS patients. Oral appliance therapy is generally considered a lifelong treatment modality. This study demonstrated that a decrease in overjet, overbite, number of occlusal contact points, and a different anterior–posterior relationship are dental changes most likely to occur. Furthermore, we found an association between the decrease in overbite and the amount of mandibular protrusion associated with wearing an oral appliance. Moreover, there was a tendency towards a mesiocclusion after 2 years of oral appliance therapy compared to CPAP therapy. In the oral appliance group, we also found a tendency towards the development of a (bi)lateral crossbite in the (pre)molar region. In the oral appliance group, more patients had a shift in molar and cuspid occlusion from class I to class III or a shift from class II to class I or III compared to the CPAP group.

Changes in overbite, overjet, and anterior–posterior relationship of the occlusion as a result of long-term oral appliance therapy have also been described in previous studies [16, 18, 19, 21, 28]. It is generally hypothesized that these changes can be attributed to a labially directed force to the mandibular incisors and a palatally directed force to the maxillary incisors during oral appliance therapy while the mandible attempts to return to a more dorsal position. However, in a study of Ringqvist and co-workers [12], no significant changes in overjet and overbite were found after 2 years of oral appliance therapy. This may be explained by the differences in appliance design. The oral appliance used in our study was made of hard acrylate which covers the entire upper and lower dental arch, while in the appliance that was used by Ringqvist and co-workers, both frontal parts were not covered by acrylate, possibly resulting in less forces applied to the upper and lower incisors. This can be a possible explanation for the absence of significant changes in overjet and overbite. In a study by Ghazal and co-workers, a significant decrease in overbite but not overjet was found [25]. The appliance used in that study was a Thornton Adjustable Positioner like the one used in the present study. However, in the latter mentioned studies, only patients with mild and moderate OSAS patients were included [12, 25]. In this present study, patients with mild, moderate, and severe OSAS were included and protrusive positions of the mandible over 75 % were applied in some patients. The relationship between the amount of mandibular protrusion and efficacy of an oral appliance has previously been described [29, 30]. Therefore, a second explanation for the different findings regarding changes of the overjet and overbite could be that an oral appliance is already effective in a less protrusive position in mild and moderate OSAS patients, resulting in less severe dental side effects.

Regression analysis showed that there appears to be an association between the decrease in overbite and the extent of mandibular protrusion. This result confirms the finding of a previous cephalometric study conducted in the same patient population [11]. Both separate findings suggest that it is important to coach patients which are treated with an oral appliance, as with an adjustable appliance, there is a risk that patients advance the mandible beyond the optimal protrusive position.

Like in several other studies [16, 21, 22], we found a significant decrease in the number of occlusal contact points in the (pre)molar region in both the oral appliance group and the CPAP group. As long-term oral appliance use results in a decrease in overjet and overbite, it is conceivable that, because of the incisal guidance phenomenon, the bite tends to open in the (pre)molar region, resulting in a decrease in the number of occlusal contact points in the (pre)molar region. Martinez-Gomis and co-workers [31] also found a significant reduction in posterior occlusal contact points after 2 years of oral appliance use. This tendency, however, reversed during the period of 2–5 years of treatment. The reason for this phenomenon can be the development of a new occlusal equilibrium over time. Therefore, it seems viable that these dental changes tend to stabilize over time. In this study, we also found a decrease in the number of occlusal contact points in the CPAP group. It has recently been advocated that nasal CPAP may change craniofacial form and may alter the relationship between the dental arches [32]. A retroclination of the maxillary incisors after long-term nasal CPAP use has also been reported in the latter study. As nasal CPAP is administered through a tight-fitted (nose) mask, it could be hypothesized that the pressure of this mask results in palatally directed forces on the frontal part of the upper dental arch. The same incisal guidance phenomenon as mentioned before could therefore explain the decrease in the number of occlusal contact points in our CPAP patients. Nevertheless, none of the patients using CPAP in our study did report any changes in occlusion. In a study, published by Ueda and co-workers [33], it was suggested that in patients using an oral appliance, certain jaw exercises might help to relieve stiffness of the masticatory muscles and accelerate the repositioning of the mandible to the normal position after a night of appliance use. It furthermore may inhibit or minimize the occlusal functional changes in predisposed patients. These results may indicate the importance of such additional exercises in patients using an oral appliance that are prone to occlusal changes.

The results presented in Tables 2 and 3 suggest that there is a tendency towards a mesial shift of the molar and cuspid occlusion after long-term use of an oral appliance. This result is in agreement with previously reported findings [16, 19–21]. However, as proposed by Almeida and co-workers [21], not all occlusal changes, which could be the result of long-term oral appliance therapy, should be interpreted as unfavorable. Regarding the patients in this study, favorable changes were more likely to be class II and the unfavorable changes seem to occur more in patients with class I baseline occlusion. We furthermore found a tendency towards the development of (bi)lateral crossbites in the (pre)molar region after long-term oral appliance use. This could be explained by the fact that, with a mesial shift in occlusion, the broader part of the mandibular dental arch will occlude with the narrower part of the maxillary dental arch. These results reinforce the importance of good pre-therapeutic information to our patients, especially in patients in whom larger occlusal changes are to be expected.

In conclusion, we found that long-term oral appliance therapy and CPAP may result in dental changes in OSAS patients. However, when treating a serious and sometimes life-threatening disorder as OSAS, therapeutic efficacy should supersede the maintenance of a patients' baseline craniofacial morphology. Discontinuation of oral appliance therapy because of the development of dental side effects should only be considered in patients who are able to tolerate or accept another effective treatment modality for their OSAS. Regarding possible dental side effects that may occur, we would like to emphasize the importance of providing adequate information to the patients before commencing oral appliance therapy. At least it should be mentioned that there is a chance that the overjet and overbite will decrease which can result in a suboptimal occlusion (e.g., less occlusal contact points and a possible development of (bi)lateral crossbite in the (pre)molar region) and altered aesthetics.
